# SARM1 Promotes Photoreceptor Degeneration in an Oxidative Stress Model of Retinal Degeneration

**DOI:** 10.3389/fnins.2022.852114

**Published:** 2022-04-01

**Authors:** Luke Gibbons, Ema Ozaki, Chris Greene, Anne Trappe, Michael Carty, Judith A. Coppinger, Andrew G. Bowie, Matthew Campbell, Sarah L. Doyle

**Affiliations:** ^1^Trinity College Institute of Neuroscience, Trinity College Dublin, Dublin, Ireland; ^2^Department of Clinical Medicine, School of Medicine, Trinity College Dublin, Dublin, Ireland; ^3^Smurfit Institute of Genetics, Trinity College Dublin, Dublin, Ireland; ^4^School of Pharmacy and Biomolecular Sciences, Royal College of Surgeons in Ireland, Dublin, Ireland; ^5^School of Biochemistry and Immunology, Trinity College Dublin, Dublin, Ireland; ^6^Trinity Biomedical Sciences Institute, Trinity College Dublin, Dublin, Ireland

**Keywords:** SARM1, sodium iodate, retinal degeneration, photoreceptors, cell death, caspase-3

## Abstract

SARM1 (sterile alpha and armadillo motif-containing protein) is a highly conserved Toll/IL-1 Receptor (TIR) adaptor with important roles in mediating immune responses. Studies in the brain have shown that SARM1 plays a role in induction of neuronal axon degeneration in response to a variety of injuries. We recently demonstrated that SARM1 is pro-degenerative in a genetic model of inherited retinopathy. This current study aimed to characterise the effect of SARM1 deletion in an alternative model of retinal degeneration (RD) in which the retinal pigment epithelium (RPE) fragments following administration of oxidising agent, sodium iodate (NaIO_3_), leading to subsequent photoreceptor cell death. Following administration of NaIO_3_, we observed no apparent difference in rate of loss of RPE integrity in SARM1 deficient mice compared to WT counterparts. However, despite no differences in RPE degeneration, photoreceptor cell number and retinal thickness were increased in *Sarm1^–/–^* mice compared to WT counterparts. This apparent protection of the photoreceptors in SARM1 deficient mice is supported by an observed decrease in pro-apoptotic caspase-3 in the photoreceptor layer of *Sarm1^–/–^* mice compared to WT. Together these data indicate a pro-degenerative role for SARM1 in the photoreceptors, but not in the RPE, in an oxidative stress induced model of retinal degeneration consistent with its known degenerative role in neurons in a range of neurodegenerative settings.

## Highlights

– SARM1 is expressed in the neural retina, but not in the RPE/choroid of mice.
– Deletion of SARM1 has no effect on the pace of RPE degeneration in the NaIO_3_ model.
– *Sarm1*^–/–^ mice show reduced photoreceptor degeneration, markers of cell death, and retain photoreceptor segment length compared to WT counterparts.

## Introduction

Retinal degenerative diseases, such as age-related macular degeneration (AMD) and retinitis pigmentosa (RP), represent one of the leading causes for incurable blindness worldwide. Although the risk factors and disease pathogenesis may differ greatly among these various diseases a common endpoint is the death of photoreceptors (Murakami et al., 2013). As these cells are responsible for the processing of light and initiation of visual signalling, photoreceptor cell death is detrimental to visual function and is ultimately the cause of vision loss in these diseases.

Retinal pigment epithelium (RPE) atrophy and photoreceptor degeneration are key features of late-stage disease in AMD termed geographic atrophy (GA). The RPE is a monolayer of pigmented cells separating the neuroretina and the choroid. It is of neuroectodermal origin and is therefore considered part of the retina. It plays a vital role in providing support for the photoreceptors and participates in the visual cycle, as such photoreceptor cell death occurs following RPE loss/dysfunction. Systemic administration of the oxidising agent sodium iodate (NaIO_3_) in mice has been demonstrated to be an effective model for studying RPE atrophy *in vivo*, as in response to NaIO_3_, RPE integrity is lost leading to subsequent secondary photoreceptor degeneration (Kiuchi et al., 2002). In this model it has been shown that RPE cells undergo cell death *via* necroptosis, an inflammatory form of regulated cell death, whereas the photoreceptor cells undergo apoptosis both as a direct effect of NaIO_3_ administration and from the loss of the supporting RPE (Wang et al., 2014; Hanus et al., 2016).

SARM1 is a member of the Toll/IL-1 Receptor (TIR) domain containing superfamily of proteins and has a role in regulating signalling pathways downstream of Toll Like Receptors (TLRs; O’Neill et al., 2003; Carty et al., 2006). However, unlike other mammalian TIR proteins, SARM1 has a novel role in promoting degeneration of axons in injured neurons, with deletion of SARM1 leading to neuroprotection in mouse and Drosophila models of neurodegeneration (Osterloh et al., 2012). SARM1 activation has been implicated in a range of models of pathological neurodegeneration, including amyotrophic lateral sclerosis (ALS; White et al., 2019), traumatic brain injury (Marion et al., 2019), and chemotherapy induced peripheral neuropathy (Geisler et al., 2016; Bosanac et al., 2021).

This pro-degenerative function is mediated *via* an enzymatic NAD^+^ cleavage site in the TIR domain of SARM1 (Gerdts et al., 2015; Essuman et al., 2017). Upon activation SARM1 consumes large amounts of NAD^+^, an essential metabolite, leading to metabolic collapse and subsequent cell death/degeneration (Essuman et al., 2017). Such activation occurs in response to a variety of cellular insults, for example mitochondrial toxins, oxygen-glucose deprivation, trophic factor withdrawal, and injury. The precise mechanism by which these disparate factors trigger SARM1 activation is not fully understood (Kim et al., 2007; Tuttolomondo et al., 2009; Mukherjee et al., 2013; Summers et al., 2014), however, recent evidence has suggested that SARM1 acts as a metabolic sensor, becoming activated when the NMN:NAD^+^ ratio within a cell becomes skewed toward the former, leading to consumption of the latter by activated SARM1 (Zhao et al., 2019; Figley et al., 2021).

Recently we identified a role for SARM1 in photoreceptor cell death, consistent with its pro-degenerative function in other neuronal cell types, in a mouse model of the inherited retinopathy retinitis pigmentosa (Ozaki et al., 2020). Here we demonstrate the pro-degenerative role of SARM1 in photoreceptors is maintained in the NaIO_3_ model of retinal degeneration. Interestingly, the attenuation of photoreceptor cell death in the absence of SARM1 occurs despite the pace of RPE atrophy remaining apparently unaffected by the presence or absence of SARM1.

## Materials and Methods

### NaIO_3_ Model of Retinal Degeneration

All studies carried out in the Smurfit Institute of Genetics in TCD adhere to the principles laid out by the internal ethics committee at TCD, and all relevant national licences were obtained before commencement of all studies. Before experiments, all mice were kept on a 12-h light/dark cycle. Mice used were C57BL/6J mice and *Sarm1*^–/–^ mice at 6–12 weeks old. NaIO_3_ (40 mg/kg or 50 mg/kg) in 0.9% saline was administered to C57BL/6J and *Sarm1*^–/–^ mice *via* a single intravenous injection (*via* tail vein). Control mice received an intravenous injection of 0.9% saline. Mice were euthanized at either 8 h, 3 days, or 7 days post injection of NaIO_3_.

### Optical Coherence Tomography and Fundus Imaging

Spectral domain optical coherence tomography (SD-OCT) and bright-field live fundus imaging was performed on mice using the image-guided OCT system (Micron IV, Phoenix Research Laboratories) and Micron Reveal software. Mouse pupils were dilated with 1% tropicamide and 2.5% phenylephrine and mice were anaesthetized using a mixture of ketamine/medetomidine (100/0.25 mg/kg). Vidisic lubricant was applied on the cornea of the anaesthetized mice, and the eye was positioned in front of the OCT camera. 50 frames were averaged along the horizontal axis above the optic disc from each eye. The thickness from the ONL to the RPE was measured at 1080 points along the retinal section using the InSight software.

### Western Blot

Retinal tissue was lysed in RIPA lysis buffer with phosphatase and protease inhibitors (Sigma-Aldrich) and centrifuged at 15,000 *g* for 15 min. Protein lysates were resolved on a 12% polyacrylamide SDS-PAGE gel and transferred onto a PVDF membrane. Membranes were blocked for 1 h in 5% non-fat milk in Tris-buffered saline with 0.05% Tween-20 (TBST), and then incubated overnight at 4°C in primary antibody against SARM1 (1:1000, 13022, Cell Signalling Technology) and β-Actin (1:5000, Sigma-Aldrich). Membranes were washed three times with TBST and incubated with horseradish peroxidase-conjugated anti-rabbit or anti-mouse antibodies (1:2000, Sigma-Aldrich) in 5% non-fat milk in TBST for 1 h at room temperature. Membranes were washed three times with TBST. Membranes were developed using enhanced chemiluminescence (Advansta) with a Fujifilm LAS3000.

### Mass Spectrometry

Retinas were lysed in 10 mM Tris-HCL, pH 7.4, 1 mM EDTA, 0.5% Triton X-100, 1 mM phenylmethylsulfonyl fluoride. Retinal samples were dissolved in 8 M urea, 100 mM Tris pH 8.5. Proteins were reduced with DTT and alkylated with IAA. Protein digestion was performed by overnight digestion with trypsin sequencing grade (Promega) resuspended in diluted TFA and stored at 4°C until MS analysis, where 10–20 μg of protein were ran on a Thermo Scientific Q Exactive mass spectrometer operated in positive ion mode and connected to a Dionex Ultimate 3000 (RSLCnano) chromatography system. All data was acquired while operating in automatic data dependent switching mode. A high-resolution (70,000) MS scan (300–1600 m/z) was performed to select the 12 most intense ions prior to MS/MS analysis using high-energy collision dissociation (HCD). Proteins were identified and quantified by MaxLFQ (Cox et al., 2014), termed MaxLFQ by searching with the MaxQuant version 1.5 against a human reference proteome database.

Modifications included C carbamlylation (fixed) and M oxidation (variable).

### RT-qPCR Analysis

Total RNA was extracted from mouse retinas using Isolate II RNA extraction kit (Bioline) as per the manufacturer’s instructions. RNA was reverse transcribed using MMLV Reverse Transcriptase (Promega). Target genes were amplified by real-time PCR with SensiFast SYBR Green (Bioline) using the ABI 7900HT system (Applied Biosystems). The comparative CT method was used for relative quantification after normalisation to the “housekeeping” gene Ubiquitin C. Primers used were as follows:

**Table:** 

**HMOX1**	Forward	5′ – GAGCCTGAATCGAGCAGAAC
	Reverse	5′ – CCTTCAAGGCCTCAGACAAA
**UBC**	Forward	5′ – CCCAGTGTTACCACCAAGAAG
	Reverse	5′ – CCCCATCACACCCAAGAACA

### Haematoxylin and Eosin Staining

NaIO_3_ (50 mg/kg) was administered to C57BL/6J and *Sarm1^–/–^* mice *via* a single intravenous injection (*via* tail vein). Mice were euthanized 3- or 7-days post injection of NaIO_3_, eyes were enucleated and fixed in Davidson’s fixative for 24 h at 4°C, followed by 3 washes with PBS. Eyes were processed in a tissue processor under gentle agitation as follows: 70% ethanol for 1 h, 80% ethanol for 1 h, 95% ethanol for 1 h, 100% ethanol for 1 h, 100% ethanol for 1 h, 50% ethanol/xylene mix for 1 h, xylene for 1 h, xylene for 1 h, paraffin at 60°C for 1 h, and paraffin under vacuum at 60°C for 1 h. Eyes were then embedded in paraffin and 5 μm sections were collected onto Polysine slides using a microtome. Sections were deparaffinized by dipping ten times in Histo Clear (National Diagnostics). Followed by rehydration by ten dips each into 100, 90, and 70% ethanol. The slides were incubated in Haematoxylin solution for 6 min at room temperature, and subsequently rinsed in cold water, and incubated in Eosin solution for 3 min and rinsed again in water. Sections were dehydrated by tens dips each into 70, 90, and 100% ethanol and once into Histoclear. Slides were mounted onto coverslips using Sub-X mounting medium (VWR) and analysed under a light microscope (Olympus 1×81). Photoreceptor degeneration was quantified by counting the number of nuclei per row in the outer nuclear layer (ONL), by counting the number of nuclei in the ONL in an area of height 450 pixels in the centre of the ONL in each image, measuring the area of the ONL using ImageJ.

### Immunohistochemistry

NaIO_3_ (50 mg/kg) was administered to C57BL/6J and *Sarm1^–/–^* mice *via* a single intravenous injection (*via* tail vein). Mice were euthanized 3- or 7-days post injection of NaIO_3_, eyes were enucleated and fixed in 4% paraformaldehyde for 1.5 h at room temperature. Eyes were washed 3 times with PBS. The lens and cornea were removed. Eyes were placed in 20% sucrose for 1 h at 4°C, followed by 30% sucrose overnight at 4°C. Eyes were embedded in OCT and frozen. 12 μm sections were collected onto Polysine slides (VWR) using a cryostat. Cryosections were blocked and permeabilized with 5% normal goat serum (NGS) (Sigma) and 0.05% Triton-X100 (Sigma) in PBS for 1 h at room temperature. Cryosections were incubated with primary antibody diluted in 5% NGS and 0.05% Triton-X100 in PBS overnight at 4°C in a humidity chamber. Primary antibody was cleaved caspase-3 (1:100, CST) and peanut agglutinin (PNA)-Alexa-568 (1:300; Invitrogen). Following three washes with PBS, cryosections were incubated with secondary antibody (AlexaFluor goat-anti rabbit 594, 1:500) for 2 h at room temperature. Cryosections were counterstained with Hoechst 33342 (1:10,000, Sigma) and washed 3 times with PBS. Slides were mounted onto coverslips with Hydromount (VWR) mounting medium and analysed using a confocal microscope (Zeiss LSM 710).

### Terminal Deoxynucleotidyl Transferase dUTP Nick End Labelling Staining

Paraffin embedded sections from WT and *Sarm1^–/–^* mice were stained using *in situ* Cell Death Detection kit, TMR red (Roche) for 1 h at 37°C according to the manufacturer’s protocol, and nuclei were stained with Hoechst 33342 (1:10,000, Sigma). Slides were mounted onto coverslips with Hydromount (VWR) mounting medium and analysed using a confocal microscope (Zeiss LSM 710). The number of TUNEL positive nuclei in the ONL were counted at 3 points across the retina.

## Results

### SARM1 Has No Effect on the Pace of Retinal Pigment Epithelium Degradation in the NaIO_3_ Model of Retinal Degeneration

**FIGURE 1:**
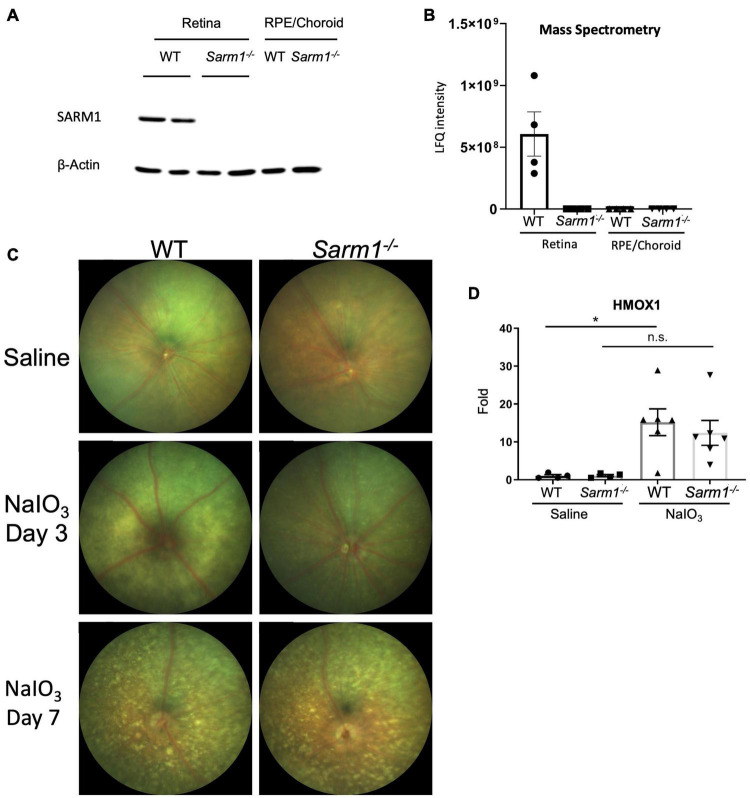
SARM1 is expressed in the retina, not the RPE/choroid and has no effect on the pace of RPE degradation in the NaIO_3_ model of retinal degeneration. Western blot analysis of SARM1 expression in the neural retina and RPE/choroid in C57BL/6J and *Sarm1*^–/–^ mice, Beta-actin is the loading control **(A)**. Mass spectrometry analysis of SARM1 expression in the neural retina and RPE/choroid in C57BL/6J and *Sarm1*^–/–^ mice, expression measured as label free quantification (LFQ) **(B)**. Colour fundus photographs of C57BL/6J and *Sarm1*^–/–^ mice 3-days and 7-days following administration of saline or NaIO_3_ (50 mg/kg) **(C)**. RT-qPCR analysis of HMOX1 transcript levels in the retina 8-h following administration of NaIO_3_ or saline (*P ≤ 0.05, by one-way ANOVA, *n* = 4 saline injected mice, *n* = 6 NaIO_3_ injected mice) **(D)**.

Given the importance of RPE function for photoreceptor health, and roles for SARM1 in non-neuronal cells (Strauss, 2005; Panneerselvam et al., 2013; Carty et al., 2019) we were interested to examine the effect of SARM1 deficiency on the RPE in a model of retinal degeneration that is thought to initiate with RPE loss. We have previously shown that the majority of SARM1 expressed in the neural retina is found in the photoreceptors. SARM1 mRNA was also detected by qPCR in the RPE/choroid of C57BL/6J mice, however, SARM1 protein expression in the RPE was not examined (Ozaki et al., 2020). We examined the expression of SARM1 protein in the mouse RPE/choroid tissue and neural retina. SARM1 was expressed in the neural retina, but was not present in the RPE when determined by Western blot (Figure 1A). This pattern of expression was confirmed by mass spectrometry analysis (Figure 1B). Consistent with the lack of SARM1 protein expression in the RPE/choroid, we observed no difference in RPE atrophy in the presence or absence of SARM1 following administration of NaIO_3_. Fundus images taken at 3 and 7 days post administration of NaIO_3_ showed no difference in the extent of pigmentary disruption between WT and *Sarm1*^–/–^ mice (Figure 1C). Additionally, there was similar upregulation of heme oxygenase 1 (HMOX1) in the retina in response to NaIO_3_, in both WT and *Sarm1*^–/–^ mice at 8 h post administration, indicating *Sarm1*^–/–^ mice experience a similar level of oxidative stress to WT mice (Figure 1D).

### SARM1 Promotes Photoreceptor Cell Death in the NaIO_3_ Model of Retinal Degeneration

**FIGURE 2:**
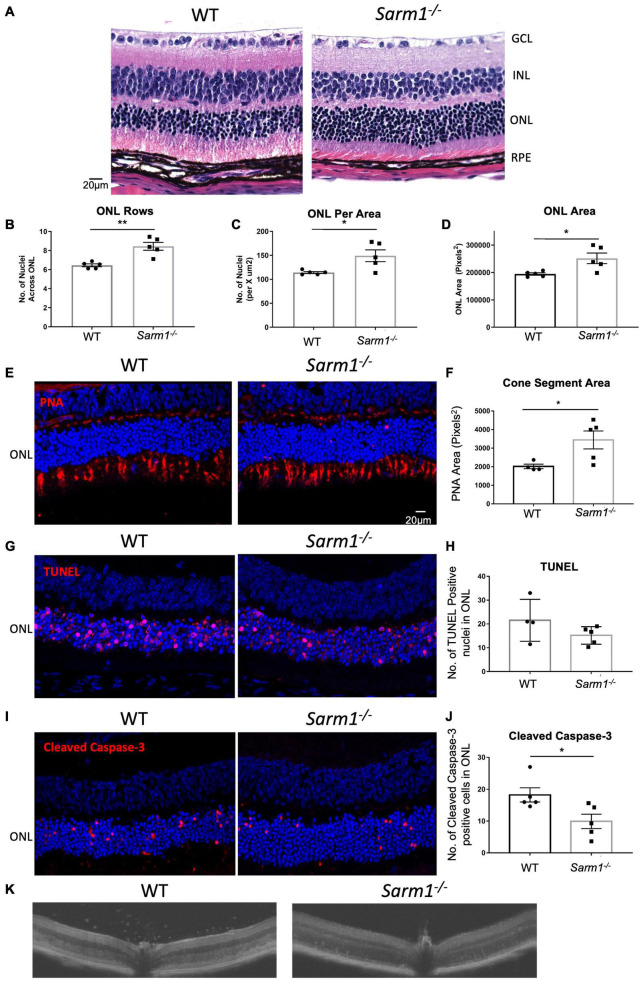
SARM1 promotes photoreceptor cell death in the NaIO_3_ model of retinal degeneration. Representative images of paraffin embedded retinal sections from WT and *Sarm1*^–/–^ mice 3-days post administration of NaIO_3_ stained with haematoxylin and eosin (H&E) **(A)**. Quantification of the number of nuclei per ONL row **(B)**, number of nuclei in the ONL in an area of height 450 pixels in the centre of the ONL in each image **(C)**, and ONL area (*P ≤ 0.05, **P ≤ 0.01, *n* = 5 mice) **(D)**. Representative images of retinal cryosections stained with PNA **(E)**. Quantification of area of PNA stained cone segments (*P ≤ 0.05, *n* = 4 WT mice, *n* = 5 *Sarm1*^–/–^ mice) **(F)**. Representative images of paraffin embedded retinal sections from WT and *Sarm1*^–/–^ mice 3-days post administration of NaIO_3_ stained with TUNEL (Red) and DAPI (Blue) **(G)**. Quantification of the number of TUNEL positive nuclei in the ONL (*n* = 4 WT mice, *n* = 5 *Sarm1*^–/–^ mice) **(H)**. Representative images of retinal cryosections from WT and *Sarm1*^–/–^ mice 3-days post administration of NaIO_3_ stained with cleaved caspase-3 (Red) and DAPI (Blue) **(I)**. Quantification of the number of cleaved caspase-3 positive cells in the ONL (*P ≤ 0.05, *n* = 4 WT mice, *n* = 5 *Sarm1*^–/–^ mice) **(J)**. Optical coherence tomography (OCT) images taken *in vivo* from C57BL/6J and *Sarm1*^–/–^ mice 3-days post administration of NaIO_3_ (50 mg/kg) **(K)**.

Examination of retinal tissue sections from WT and *Sarm1*^–/–^ mice at 3-days post administration of NaIO_3_, stained with haematoxylin and eosin (H&E) (Figure 2A), supported the observation by fundus imaging that the RPE is affected to a similar extent in WT (Figure 2A, left hand panel) and *Sarm1*^–/–^ mice (Figure 2A, right hand panel). However, H&E staining also showed that there are more photoreceptor nuclei in *Sarm1*^–/–^ mice. We observed that there are significantly more nuclei in ONL rows present in *Sarm1*^–/–^ at 3 days post administration of NaIO_3_ compared to WT counterparts (Figure 2B). Similarly, the number of ONL nuclei in a set area at the central posterior region of each section (Figure 2C) and the area of the ONL (Figure 2D) are significantly increased in the absence of SARM1. Furthermore, PNA staining in the sub-retinal space indicates significantly more cone photoreceptor segments remain in *Sarm1*^–/–^ mice compared with WT mice post administration of NaIO_3_ (Figures 2E,F).

SARM1 mediated neuronal degeneration does not require caspase-3 or RIPK3 to initiate cell death, however, it has been reported that photoreceptor cells undergo apoptosis following administration of NaIO_3_ (Hanus et al., 2016). In order to determine whether loss of SARM1 affects photoreceptor apoptosis we utilised two markers of cell death: Terminal deoxynucleotidyl transferase dUTP nick end labelling (TUNEL) and cleaved caspase-3. We observed no significant difference in the number of TUNEL positive nuclei in the ONL of *Sarm1*^–/–^ mice compared to WT counterparts (Figures 2G,H). However, there are significantly higher amounts of cleaved caspase-3 positive nuclei in the ONL of WT mice, compared to *Sarm1*^–/–^ mice, indicating decreased levels of photoreceptor apoptosis in the absence of SARM1 at this timepoint (Figures 2I,J). Additionally, there is an increased number of immune cells infiltrating into the retina in WT mice compared to *Sarm1*^–/–^ mice at 3 days following administration of NaIO_3_, as observed by OCT imaging (Figure 2K). Infiltrating cells were most abundant in WT mice around the optic nerve head and were present above the retinal ganglion cell layer of the retina (Figure 2K). These data indicate that SARM1, among other cell death pathways, is promoting photoreceptor cell death in response to NaIO_3_ administration, and that deletion of SARM1 can reduce these observed effects.

### Loss of SARM1 Preserves Photoreceptor Number and Length in the NaIO_3_ Model of Retinal Degeneration, Following Severe Retinal Pigment Epithelium Degradation

**FIGURE 3:**
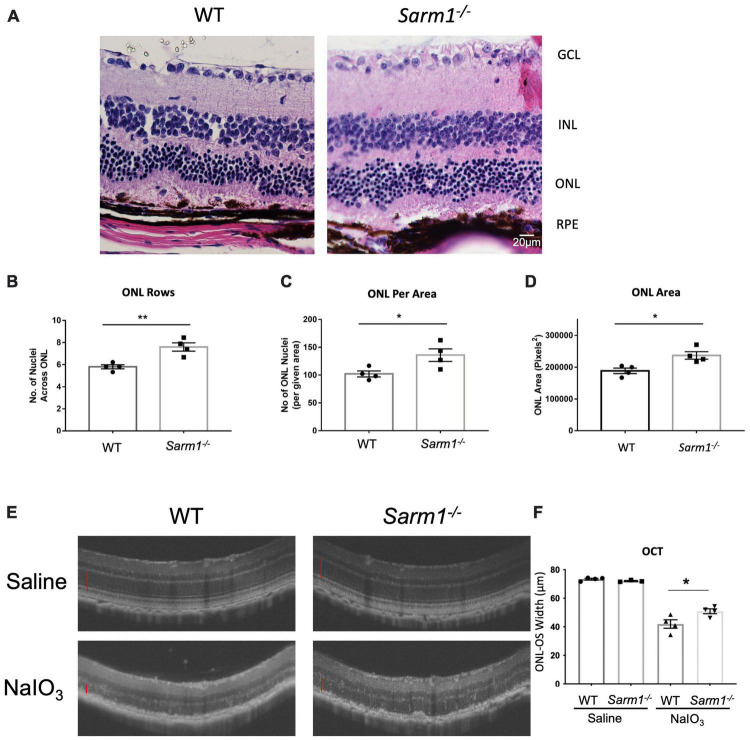
Loss of SARM1 preserves photoreceptor number and length in the NaIO_3_ model of retinal degeneration, following severe RPE degradation. Representative images of paraffin embedded retinal sections from WT and *Sarm1^–/–^* mice 7-days post administration of NaIO_3_ stained with haematoxylin & eosin (H&E) **(A)**. Quantification of the number of nuclei per ONL row **(B)**, number of nuclei in the ONL in an area of height 450 pixels in the centre of the ONL in each image **(C)**, and ONL area **(D)** (*P ≤ 0.05, **P ≤ 0.01, *n* = 4 WT NaIO_3_ mice, *n* = 4 *Sarm1^–/–^* NaIO_3_ mice). Optical coherence tomography (OCT) images taken *in vivo* from C57BL/6J and *Sarm1^–/–^* mice 7-days post administration of NaIO_3_ (50 mg/kg) **(E)**. Quantification of the distance of the ONL to the outer segments (OS) (marked with red line on OCT images) InSight (*P ≤ 0.05, by Student’s unpaired *t* test, *n* = 4 WT saline mice, *n* = 3 *Sarm1^–/–^* saline mice, *n* = 4 WT NaIO_3_ mice, *n* = 4 *Sarm1^–/–^* mice) **(F)**.

Haematoxylin and eosin stained retinal sections (Figure 2A) indicate that the RPE appears to remain largely intact at 3 days following administration of NaIO_3_. To determine whether loss of SARM1 could confer protection against photoreceptor cell death following more severe degradation we examined H&E stained retinal sections at 7 days following administration of NaIO_3_. At 7 days following administration of NaIO_3_ there is a large degree of RPE degradation (Figure 3A), despite this there is increased photoreceptor cell survival in *Sarm1*^–/–^ mice compared to WT counterparts. Examination of H&E stained paraffin embedded retinal sections from WT and *Sarm1*^–/–^ mice at 7 days following NaIO_3_ administration shows that there are significantly more photoreceptor nuclei in the ONL, as measured by number of nuclei per row (Figure 3B), the number of ONL nuclei in an area at the centre of each section (Figure 3C), and ONL area (Figure 3D). Using optical coherence tomography (OCT) we measured the thickness between the outer plexiform layer (which forms the interface between the bipolar cells and photoreceptors) and the RPE. This measurement gives the thickness of the entire photoreceptor length, inclusive of both the cell soma and photoreceptor segments. We observed a significant decrease in photoreceptor length in WT compared to *Sarm1*^–/–^ mice at 7 days following NaIO_3_ injection (Figures 3E,F). These data indicate that the protection against photoreceptor cell death conferred by deletion of SARM1 can persist following further degradation of the supporting RPE.

## Discussion

Previous studies from our lab (Ozaki et al., 2020) and others (Sasaki et al., 2020) have shown that SARM1 has a pro-degenerative role in inherited retinopathies, using models of retinitis pigmentosa and Leber Congenital Amaurosis (LCA). In this study, we have identified a similar, previously uncharacterised role for SARM1 in a model of RPE atrophy. We show that SARM1 promotes photoreceptor cell death, but has no obvious effect on degeneration of the RPE in the NaIO_3_ model.

SARM1 has been previously shown to be highly expressed in neural tissues in comparison to other tissue types. Here, our data clearly demonstrates that in the eye SARM1 is expressed in the neural retina, but not in the RPE/choroid. As such, in the context of the NaIO_3_ model, in which there is initially loss of RPE integrity followed by subsequent photoreceptor cell degeneration, any alterations to photoreceptor cell degeneration in *Sarm1*^–/–^ mice are likely to be a direct consequence of loss of SARM1 in photoreceptor cells, rather than a direct effect on the supporting RPE. Indeed, SARM1 deficiency did not appear to slow the rate of pigmentary disruption as observed by fundus photography, and there was no apparent difference in the fragmentation of the RPE monolayer when observed by H&E staining in tissue sections at either timepoint post treatment with NaIO_3_. Despite this balance in the acute loss of RPE integrity in both WT and *Sarm1*^–/–^ mice, it is clear that SARM1 deficiency is protective for photoreceptor cells, as *Sarm1*^–/–^ mice demonstrate a decreased rate of thinning of the retina and reduced loss of photoreceptor cell nuclei.

The precise mechanism by which SARM1 promotes cell death in photoreceptors in this model remains to be described. We have previously shown that SARM1 cleaves NAD^+^ in photoreceptor cells in a model of RP, and it is likely that a similar mechanism is at play here (Ozaki et al., 2020). This is also the mechanism observed in axonal degeneration of peripheral neurons (Gerdts et al., 2015; Essuman et al., 2017). However, it is clear that there is reduced cleaved caspase-3, a marker of apoptosis, in the photoreceptors in SARM1 deficient mice. This may be a consequence of SARM1 deficiency reducing extrinsic death ligands or intrinsic stress signalling and subsequently reducing activation of caspase-3, or it may indicate a role for caspase-3 in SARM1-mediated photoreceptor degeneration, akin to that described for SARM1- induction of mitochondrial apoptosis in activated CD8 T cells (Panneerselvam et al., 2013). Furthermore, in addition to loss of RPE trophic support, photoreceptor degeneration in the NaIO_3_ model may be triggered by other factors, including direct effects of oxidising agent NaIO_3_ on photoreceptors, and immune cell infiltration (Wang et al., 2014; Moriguchi et al., 2018). Indeed, SARM1 has been shown to induce chemokines CCL2, CCL7, and CCL12 in a model of peripheral nerve injury (Wang et al., 2018), these are distinct from the passenger mutations in Ccl3/4/5 carried by the *Sarm1*^–/–^ mice (Uccellini et al., 2020) and interestingly, are key chemokines involved in mononuclear cell infiltration in the retina (Natoli et al., 2016; Karlen et al., 2018). In this study, we also observed reduced immune cell infiltrate when assessed by OCT, which may account for some aspect of the delayed photoreceptor degeneration in the *Sarm1*^–/–^ mice. However, it is not clear whether the cell infiltration is lessened in response to the reduced numbers of dying cells and consequent reduced inflammatory environment or is due to a direct effect of loss of chemokine signalling due to SARM1 deficiency. As such further investigation is required to fully describe the mechanism by which SARM1 becomes activated in this model and by which it promotes photoreceptor degeneration.

To date a number of studies have been published describing various molecules, such as zinc chloride and isoquinolines, that are capable of inhibiting the NAD^+^ cleavage activity of SARM1, through a proposed mechanism involving interaction and possible modification of cysteine residues (Loring et al., 2020; Bosanac et al., 2021). These various inhibitors have been shown to prevent axonal degeneration induced by SARM1 following injury (Hughes et al., 2021) or in response to paclitaxel (Bosanac et al., 2021), both *in vitro* and *in vivo*. Our study adds to the growing body of data indicating that SARM1 inhibitors may also be of use in retinal degenerative diseases that have a SARM1 dependent component (Ozaki et al., 2020; Sasaki et al., 2020) making SARM1 a potential therapeutic target of interest for future study in retinal degeneration.

## Data Availability Statement

The original contributions presented in the study are included in the article/supplementary material, further inquiries can be directed to the corresponding author.

## Ethics Statement

The animal study was reviewed and approved by Trinity College AREC.

## Author Contributions

LG, EO, CG, AT, and JC performed experiments. LG, EO, AB, and MC participated in the design of the study. SD conceived and designed the study. LG, EO, and SD wrote the manuscript. All authors read and approved the final manuscript.

## Conflict of Interest

The authors declare that the research was conducted in the absence of any commercial or financial relationships that could be construed as a potential conflict of interest.

## Publisher’s Note

All claims expressed in this article are solely those of the authors and do not necessarily represent those of their affiliated organizations, or those of the publisher, the editors and the reviewers. Any product that may be evaluated in this article, or claim that may be made by its manufacturer, is not guaranteed or endorsed by the publisher.

## Abbreviations

ALS: amyotrophic lateral sclerosis
AMD: age related macular degeneration
GA: geographic atrophy
H&E: haematoxylin and eosin
HMOX1: heme oxygenase 1
NaIO3: sodium iodate
ONL: outer nuclear layer
PNA: Peanut agglutinin
RP: retinitis pigmentosa
SARM1: sterile alpha and toll/interleukin-1 receptor motif–containing 1
SD-OCT: spectral domain optical coherence tomography
TIR: toll-IL-1 receptor
TLR: toll like receptor
TUNEL: terminal deoxynucleotidyl transferase dUTP nick end labelling.
